# 4-(1*H*-Benzimidazol-2-ylmeth­oxy)-3-meth­oxy­benzaldehyde tetra­hydrate

**DOI:** 10.1107/S1600536811027164

**Published:** 2011-07-13

**Authors:** Jerry P. Jasinski, James A. Golen, S. Samshuddin, B. Narayana, H. S. Yathirajan

**Affiliations:** a Department of Chemistry, Keene State College, 229 Main Street, Keene, NH 03435-2001, USA; b Department of Studies in Chemistry, Mangalore University, Mangalagangotri 574 199, India; c Department of Studies in Chemistry, University of Mysore, Manasagangotri, Mysore 570 006, India

## Abstract

In the title compound, C_16_H_14_N_2_O_3_·4H_2_O, the dihedral angle between the mean planes of the benzimidazole ring system and benzene ring is 2.9 (1)°. The aldehyde group is disordered over two sets of sites with refined occupancies of 0.559 (4) and 0.441 (4). In the crystal, extensive inter­molecular O—H⋯O, O—H⋯N and N—H⋯O hydrogen bonds in concert with weak π–π stacking inter­actions [centroid–centroid distances = 3.6104 (9), 3.6288 (9) and 3.9167 (10) Å] create a three-dimensional network.

## Related literature

For the pharmaceutical and biological activity of benzimidazole compounds, see: Pujar *et al.* (1988 ▶); Bouwman *et al.* (1990 ▶). For plant-protective agents in the field of pest control, see: Madkour *et al.* (2006 ▶). For related structures, see: Akkurt *et al.* (2011 ▶); Jian *et al.* (2003 ▶); Jasinski *et al.* (2010 ▶, 2011 ▶); Odabaşoğlu *et al.* (2007) ▶. For standard bond lengths, see: Allen *et al.* (1987 ▶).
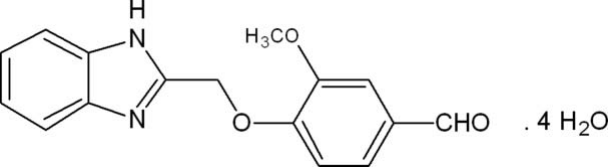


## Experimental

### 

#### Crystal data

C_16_H_14_N_2_O_3_·4H_2_O
*M* *_r_* = 354.36
Triclinic, 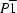

*a* = 6.8953 (6) Å
*b* = 11.4266 (13) Å
*c* = 11.7287 (14) Å
α = 107.965 (10)°
β = 90.906 (8)°
γ = 91.769 (8)°
*V* = 878.32 (16) Å^3^
*Z* = 2
Mo *K*α radiation
μ = 0.11 mm^−1^
*T* = 173 K
0.35 × 0.33 × 0.20 mm

#### Data collection

Oxford Diffraction Xcalibur Eos Gemini diffractometer
Absorption correction: multi-scan (*CrysAlis RED*; Oxford Diffraction, 2010 ▶)’ *T*_min_ = 0.964, *T*_max_ = 0.979
8245 measured reflections
4539 independent reflections
3499 reflections with *I* > 2σ(*I*)
*R*_int_ = 0.016

#### Refinement

*R*[*F* ^2^ > 2σ(*F* ^2^)] = 0.049
*wR*(*F* ^2^) = 0.152
*S* = 1.02
4539 reflections
273 parameters
17 restraints
H atoms treated by a mixture of independent and constrained refinement
Δρ_max_ = 0.25 e Å^−3^
Δρ_min_ = −0.36 e Å^−3^

### 

Data collection: *CrysAlis PRO* (Oxford Diffraction, 2010 ▶); cell refinement: *CrysAlis PRO*; data reduction: *CrysAlis RED* (Oxford Diffraction, 2010 ▶); program(s) used to solve structure: *SHELXS97* (Sheldrick, 2008 ▶); program(s) used to refine structure: *SHELXL97* (Sheldrick, 2008 ▶); molecular graphics: *SHELXTL* (Sheldrick, 2008 ▶); software used to prepare material for publication: *SHELXTL*.

## Supplementary Material

Crystal structure: contains datablock(s) global, I. DOI: 10.1107/S1600536811027164/lh5279sup1.cif

Structure factors: contains datablock(s) I. DOI: 10.1107/S1600536811027164/lh5279Isup2.hkl

Supplementary material file. DOI: 10.1107/S1600536811027164/lh5279Isup3.cml

Additional supplementary materials:  crystallographic information; 3D view; checkCIF report

## Figures and Tables

**Table 1:** Hydrogen-bond geometry (Å, °)

*D*—H⋯*A*	*D*—H	H⋯*A*	*D*⋯*A*	*D*—H⋯*A*
O4—H4*WB*⋯O5	0.84 (2)	1.98 (2)	2.809 (2)	170 (2)
O4—H4*WA*⋯O5^i^	0.87 (2)	2.04 (2)	2.880 (2)	162 (2)
O5—H5*WB*⋯N2	0.85 (1)	1.96 (2)	2.8003 (16)	171 (2)
O5—H5*WA*⋯O7^ii^	0.85 (2)	1.94 (2)	2.7882 (19)	171 (2)
O6—H6*WA*⋯O4^iii^	0.85 (2)	2.16 (2)	2.996 (2)	171 (3)
O6—H6*WB*⋯O2	0.85 (2)	2.40 (2)	3.187 (3)	154 (3)
O7—H7*WB*⋯O4^iv^	0.84 (2)	2.01 (2)	2.844 (2)	174 (2)
O7—H7*WA*⋯O3*A*	0.81 (2)	1.98 (2)	2.721 (3)	151 (2)
N1—H1*N*⋯O6	0.83 (1)	2.02 (1)	2.8152 (19)	160 (2)

Symmetry codes: (i) 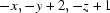; (ii) 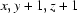; (iii) 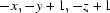; (iv) 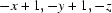.

**Fig. 1.**
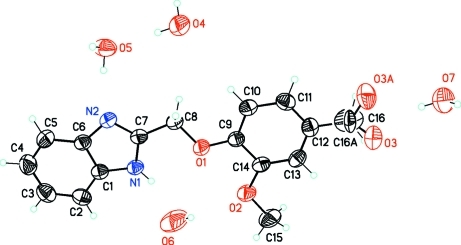
The molecular structure of the title compound showing 50% probability displacement ellipsoids.

**Fig. 2.**
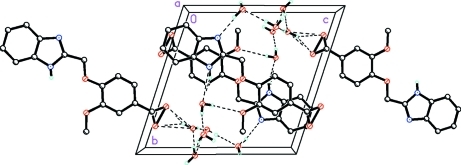
Packing diagram of the title compound viewed along the a axis. Dashed lines represent O—H···O, O—H···N and N—H···O hydrogen bonds.

**Fig. 3.**
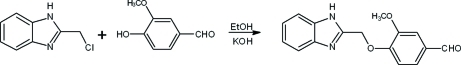
Reaction scheme of the title compound, (I).

## supplementary crystallographic information

### Comment

The benzimidazole ring system and its related compounds play an important role in pharmaceutical and agricultural fields due to their broad spectrum of biological activities (Pujar *et al.*, 1988,  Bouwman *et al.*, 1990).  The synthesis of novel benzimidazole derivatives remains a main focus of medicinal research. Benzimidazoles are also useful as insecticides, acaricides, nematocides, herbicides and other plant-protective agents in the field of pest control (Madkour *et al.*, 2006).  In addition, benzimidazole derivatives have played a crucial role in the theoretical development of heterocyclic chemistry and are also used extensively in organic synthesis.  The crystal structures of some benzimidazole derivatives viz., 2-chloromethyl-1H-benzimidazole nitrate (Jian *et al.*, 2003) and 5-methoxy-1H-benzo[d]imidazole-2(3H)-thione (Odabaşoğlu *et al.*, 2007) have been reported. In continuation of our work on the synthesis of benzimidazole containing aldehydes and their chalcones (Jasinski *et al.*, 2010, 2011; Akkurt *et al.*, 2011) and in view of the importance of benzimidazoles, the title compound, (I), was synthesized and its crystal structure is reported herein.

The molecular structure of the title compound is shown in Fig. 1. In (I) the dihedral angle between the mean planes of the benzimidazole ring system and benzene ring is 2.9 (1)°. The aldehyde group is disordered over two sets of sites corresponding to a rotation of approximately 180° about the C12-C16 bond with refined occupancies of 0.441 (4) and 0.559 (4).  Bond distances are in normal ranges (Allen *et al.*, 1987).  Extensive O—H···O, O—H···N and N—H···O hydrogen bonds (Table 1) in concert with weak π–π stacking interactions (Table 2) create a 3-D network (Fig. 2).

### Experimental

Vanillin (1.52g, 0.01 mole) was dissolved in 30 mL of ethanolic KOH (0.56g, 0.01 mole) and the solution was stirred for 1 h. 2-chloromethyl-1H-benzimidazole (1.66g, 0.01 mole) was added with continuous stirring and refluxed for 5 h (Fig. 3).  The reaction mixture was cooled to room temperature and poured into crushed ice.  The solid products that separated out were filtered off and recrystallized in ethanol. Single crystals were grown from ethanol by the slow evaporation method which yielded the tetrahydrate of the product (m.p.: 381-382 K) with an yield of 46%.

### Refinement

The N–H atom was located in a difference Fourier map and refined isotropically with DFIX = 0.86Å.  The O–H atoms were also located in differnce Fourier maps and refined isotropically with DFIX = 0.84Å and DANG = 1.35Å. DFIX and DANG commands are in the SHELXL (Sheldrick, 2008) software. The C and O atoms on the aldehyde group were refined as disordered over two sets of sites for C16/C16A and O3/O3A [occupancy ratio 0.441 (4):0.559 (4)]. All of the remaining H atoms were placed in calculated positions and refined using a riding-model approximation with C—H lengths of 0.95Å (CH), 0.99Å (CH_2_) or 0.98Å (CH_3_). The isotropic displacement parameters for these atoms were set to 1.19-1.21 (CH, CH_2_ ) or 1.50 (CH_3_) times *U*_eq_ of the parent atom.

### Crystal data

**Table:**

C_16_H_14_N_2_O_3_·4H_2_O	*Z* = 2
*M**_r_* = 354.36	*F*(000) = 376
Triclinic, *P*1	*D*_x_ = 1.340 Mg m^−^^3^
Hall symbol: -P 1	Mo *K*α radiation, λ = 0.71073 Å
*a* = 6.8953 (6) Å	Cell parameters from 4026 reflections
*b* = 11.4266 (13) Å	θ = 3.4–32.3°
*c* = 11.7287 (14) Å	µ = 0.11 mm^−^^1^
α = 107.965 (10)°	*T* = 173 K
β = 90.906 (8)°	Block, pale yellow
γ = 91.769 (8)°	0.35 × 0.33 × 0.20 mm
*V* = 878.32 (16) Å^3^	

### Data collection

**Table:**

Oxford Diffraction Xcalibur Eos Gemini diffractometer	4539 independent reflections
Radiation source: Enhance (Mo) X-ray Source	3499 reflections with *I* > 2σ(*I*)
graphite	*R*_int_ = 0.016
Detector resolution: 16.1500 pixels mm^-1^	θ_max_ = 28.7°, θ_min_ = 3.4°
ω scans	*h* = −9→6
Absorption correction: multi-scan (*CrysAlis RED*; Oxford Diffraction, 2010)'	*k* = −15→12
*T*_min_ = 0.964, *T*_max_ = 0.979	*l* = −14→15
8245 measured reflections	

### Refinement

**Table:**

Refinement on *F*^2^	Primary atom site location: structure-invariant direct methods
Least-squares matrix: full	Secondary atom site location: difference Fourier map
*R*[*F*^2^ > 2σ(*F*^2^)] = 0.049	Hydrogen site location: inferred from neighbouring sites
*wR*(*F*^2^) = 0.152	H atoms treated by a mixture of independent and constrained refinement
*S* = 1.02	*w* = 1/[σ^2^(*F*_o_^2^) + (0.0835*P*)^2^ + 0.139*P*] where *P* = (*F*_o_^2^ + 2*F*_c_^2^)/3
4539 reflections	(Δ/σ)_max_ = 0.002
273 parameters	Δρ_max_ = 0.25 e Å^−^^3^
17 restraints	Δρ_min_ = −0.36 e Å^−^^3^

### Special details

**Table:**

Geometry. All esds (except the esd in the dihedral angle between two l.s. planes) are estimated using the full covariance matrix. The cell esds are taken into account individually in the estimation of esds in distances, angles and torsion angles; correlations between esds in cell parameters are only used when they are defined by crystal symmetry. An approximate (isotropic) treatment of cell esds is used for estimating esds involving l.s. planes.
Refinement. Refinement of *F*^2^ against ALL reflections. The weighted *R*-factor *wR* and goodness of fit *S* are based on *F*^2^, conventional *R*-factors *R* are based on *F*, with *F* set to zero for negative *F*^2^. The threshold expression of *F*^2^ > σ(*F*^2^) is used only for calculating *R*-factors(gt) *etc*. and is not relevant to the choice of reflections for refinement. *R*-factors based on *F*^2^ are statistically about twice as large as those based on *F*, and *R*- factors based on ALL data will be even larger.

### Fractional atomic coordinates and isotropic or equivalent isotropic displacement parameters (Å^2^)

**Table:**

	*x*	*y*	*z*	*U*_iso_*/*U*_eq_	Occ. (<1)
O1	0.25247 (16)	0.50917 (9)	0.48675 (9)	0.0433 (3)	
O2	0.27855 (17)	0.27445 (9)	0.41320 (10)	0.0504 (3)	
O3	0.3970 (4)	0.1784 (3)	−0.0482 (2)	0.0613 (10)	0.441 (4)
O3A	0.3989 (4)	0.3155 (2)	−0.0763 (2)	0.0688 (9)	0.559 (4)
O4	0.1293 (2)	0.86262 (11)	0.38472 (13)	0.0662 (4)	
H4WB	0.161 (3)	0.8992 (19)	0.4568 (14)	0.079*	
H4WA	0.045 (3)	0.9090 (19)	0.3647 (19)	0.079*	
O5	0.18563 (19)	0.98294 (11)	0.63137 (12)	0.0599 (3)	
H5WB	0.192 (3)	0.9297 (16)	0.6684 (18)	0.072*	
H5WA	0.286 (3)	1.0304 (17)	0.6559 (18)	0.072*	
O6	0.1819 (3)	0.33687 (14)	0.6899 (2)	0.0961 (6)	
H6WA	0.101 (3)	0.276 (2)	0.674 (2)	0.115*	
H6WB	0.246 (4)	0.326 (3)	0.626 (2)	0.115*	
O7	0.4891 (2)	0.15864 (14)	−0.29388 (13)	0.0701 (4)	
H7WB	0.603 (3)	0.158 (2)	−0.3182 (19)	0.084*	
H7WA	0.493 (3)	0.192 (2)	−0.2216 (14)	0.084*	
N1	0.18875 (16)	0.59173 (10)	0.72323 (11)	0.0364 (3)	
H1N	0.192 (2)	0.5154 (12)	0.6960 (14)	0.044*	
N2	0.19911 (17)	0.78893 (10)	0.72974 (10)	0.0386 (3)	
C1	0.16508 (18)	0.65805 (12)	0.84166 (12)	0.0363 (3)	
C2	0.1383 (2)	0.62248 (15)	0.94340 (14)	0.0462 (3)	
H2A	0.1349	0.5383	0.9401	0.055*	
C3	0.1168 (2)	0.71524 (17)	1.04909 (15)	0.0549 (4)	
H3A	0.0961	0.6948	1.1207	0.066*	
C4	0.1248 (3)	0.83881 (17)	1.05371 (15)	0.0588 (4)	
H4A	0.1095	0.9004	1.1285	0.071*	
C5	0.1541 (2)	0.87414 (14)	0.95290 (14)	0.0508 (4)	
H5A	0.1614	0.9586	0.9573	0.061*	
C6	0.17285 (18)	0.78171 (12)	0.84452 (12)	0.0375 (3)	
C7	0.20690 (18)	0.67405 (11)	0.66209 (12)	0.0348 (3)	
C8	0.2316 (2)	0.63843 (12)	0.53048 (12)	0.0407 (3)	
H8A	0.1171	0.6617	0.4915	0.049*	
H8B	0.3480	0.6816	0.5122	0.049*	
C9	0.28517 (18)	0.46020 (12)	0.36761 (12)	0.0352 (3)	
C10	0.3059 (2)	0.52749 (13)	0.28790 (13)	0.0413 (3)	
H10A	0.2975	0.6145	0.3152	0.050*	
C11	0.3390 (2)	0.46721 (14)	0.16864 (13)	0.0443 (3)	
H11A	0.3551	0.5132	0.1141	0.053*	
C12	0.34859 (19)	0.34084 (14)	0.12823 (13)	0.0418 (3)	
C13	0.32778 (19)	0.27242 (13)	0.20786 (13)	0.0409 (3)	
H13A	0.3346	0.1853	0.1796	0.049*	
C14	0.29751 (18)	0.33106 (12)	0.32700 (13)	0.0375 (3)	
C15	0.2914 (3)	0.14435 (14)	0.37694 (19)	0.0631 (5)	
H15A	0.2866	0.1158	0.4476	0.095*	
H15B	0.4139	0.1212	0.3363	0.095*	
H15C	0.1825	0.1063	0.3219	0.095*	
C16	0.3765 (19)	0.2805 (10)	0.0036 (9)	0.078 (5)	0.441 (4)
H16A	0.3785	0.3337	−0.0447	0.093*	0.441 (4)
C16A	0.3838 (12)	0.2765 (6)	0.0018 (4)	0.046 (2)	0.559 (4)
H16B	0.3959	0.1900	−0.0179	0.055*	0.559 (4)

### Atomic displacement parameters (Å^2^)

**Table:**

	*U*^11^	*U*^22^	*U*^33^	*U*^12^	*U*^13^	*U*^23^
O1	0.0595 (6)	0.0317 (5)	0.0400 (5)	0.0043 (4)	0.0048 (4)	0.0129 (4)
O2	0.0665 (7)	0.0350 (5)	0.0546 (6)	0.0035 (5)	0.0063 (5)	0.0206 (4)
O3	0.0618 (18)	0.064 (2)	0.0498 (16)	0.0027 (14)	0.0093 (12)	0.0047 (14)
O3A	0.0735 (16)	0.0759 (18)	0.0524 (14)	−0.0041 (12)	0.0180 (11)	0.0130 (12)
O4	0.0898 (10)	0.0485 (7)	0.0610 (8)	0.0035 (6)	0.0078 (7)	0.0177 (6)
O5	0.0660 (8)	0.0529 (7)	0.0692 (8)	−0.0063 (6)	−0.0137 (6)	0.0329 (6)
O6	0.0934 (13)	0.0469 (8)	0.1487 (18)	−0.0067 (8)	−0.0135 (11)	0.0330 (10)
O7	0.0674 (8)	0.0725 (9)	0.0644 (8)	−0.0059 (7)	0.0001 (7)	0.0131 (7)
N1	0.0372 (6)	0.0298 (5)	0.0432 (6)	−0.0010 (4)	−0.0011 (4)	0.0131 (5)
N2	0.0406 (6)	0.0322 (5)	0.0437 (6)	0.0043 (4)	0.0011 (5)	0.0126 (5)
C1	0.0276 (6)	0.0387 (6)	0.0441 (7)	0.0016 (5)	−0.0005 (5)	0.0149 (5)
C2	0.0415 (7)	0.0535 (8)	0.0491 (8)	−0.0006 (6)	−0.0005 (6)	0.0243 (7)
C3	0.0500 (9)	0.0739 (11)	0.0443 (8)	0.0067 (8)	0.0037 (7)	0.0229 (8)
C4	0.0606 (10)	0.0666 (11)	0.0439 (8)	0.0161 (8)	0.0049 (7)	0.0078 (7)
C5	0.0540 (9)	0.0436 (8)	0.0512 (9)	0.0139 (7)	0.0024 (7)	0.0083 (7)
C6	0.0310 (6)	0.0382 (7)	0.0439 (7)	0.0058 (5)	0.0007 (5)	0.0133 (5)
C7	0.0308 (6)	0.0327 (6)	0.0430 (7)	0.0003 (5)	−0.0015 (5)	0.0149 (5)
C8	0.0506 (8)	0.0315 (6)	0.0411 (7)	0.0011 (5)	0.0005 (6)	0.0128 (5)
C9	0.0326 (6)	0.0345 (6)	0.0394 (7)	0.0016 (5)	0.0006 (5)	0.0127 (5)
C10	0.0445 (7)	0.0350 (6)	0.0470 (7)	0.0024 (5)	0.0018 (6)	0.0162 (6)
C11	0.0419 (7)	0.0502 (8)	0.0461 (8)	0.0026 (6)	0.0031 (6)	0.0225 (6)
C12	0.0310 (6)	0.0509 (8)	0.0411 (7)	0.0033 (5)	0.0037 (5)	0.0104 (6)
C13	0.0324 (6)	0.0355 (6)	0.0516 (8)	0.0024 (5)	0.0024 (6)	0.0087 (6)
C14	0.0316 (6)	0.0342 (6)	0.0490 (8)	0.0015 (5)	0.0009 (5)	0.0162 (6)
C15	0.0776 (12)	0.0351 (8)	0.0826 (13)	0.0022 (8)	0.0069 (10)	0.0266 (8)
C16	0.039 (6)	0.085 (9)	0.116 (9)	−0.005 (5)	−0.002 (5)	0.042 (7)
C16A	0.042 (4)	0.060 (4)	0.0252 (19)	0.007 (3)	0.011 (2)	−0.004 (2)

### Geometric parameters (Å, °)

**Table:**

O1—C9	1.3609 (17)	C3—H3A	0.9500
O1—C8	1.4190 (16)	C4—C5	1.378 (2)
O2—C14	1.3638 (17)	C4—H4A	0.9500
O2—C15	1.4206 (18)	C5—C6	1.390 (2)
O3—C16	1.151 (8)	C5—H5A	0.9500
O3A—C16A	1.140 (7)	C7—C8	1.4840 (19)
O4—H4WB	0.841 (15)	C8—H8A	0.9900
O4—H4WA	0.873 (15)	C8—H8B	0.9900
O5—H5WB	0.851 (14)	C9—C10	1.3880 (19)
O5—H5WA	0.853 (15)	C9—C14	1.4097 (18)
O6—H6WA	0.845 (16)	C10—C11	1.381 (2)
O6—H6WB	0.850 (16)	C10—H10A	0.9500
O7—H7WB	0.838 (15)	C11—C12	1.378 (2)
O7—H7WA	0.814 (15)	C11—H11A	0.9500
N1—C7	1.3514 (16)	C12—C13	1.397 (2)
N1—C1	1.3768 (18)	C12—C16	1.430 (9)
N1—H1N	0.832 (13)	C12—C16A	1.466 (4)
N2—C7	1.3110 (17)	C13—C14	1.373 (2)
N2—C6	1.3881 (18)	C13—H13A	0.9500
C1—C2	1.387 (2)	C15—H15A	0.9800
C1—C6	1.4023 (18)	C15—H15B	0.9800
C2—C3	1.374 (2)	C15—H15C	0.9800
C2—H2A	0.9500	C16—H16A	0.9500
C3—C4	1.396 (3)	C16A—H16B	0.9500
			
C9—O1—C8	116.72 (10)	C7—C8—H8B	110.0
C14—O2—C15	117.42 (13)	H8A—C8—H8B	108.4
H4WB—O4—H4WA	105.6 (17)	O1—C9—C10	124.95 (12)
H5WB—O5—H5WA	104.9 (17)	O1—C9—C14	114.89 (11)
H6WA—O6—H6WB	105 (2)	C10—C9—C14	120.15 (13)
H7WB—O7—H7WA	107.6 (19)	C11—C10—C9	119.63 (13)
C7—N1—C1	106.93 (11)	C11—C10—H10A	120.2
C7—N1—H1N	127.5 (11)	C9—C10—H10A	120.2
C1—N1—H1N	125.5 (11)	C12—C11—C10	120.42 (13)
C7—N2—C6	104.49 (11)	C12—C11—H11A	119.8
N1—C1—C2	132.25 (13)	C10—C11—H11A	119.8
N1—C1—C6	105.01 (11)	C11—C12—C13	120.31 (13)
C2—C1—C6	122.74 (13)	C11—C12—C16	119.2 (5)
C3—C2—C1	116.54 (14)	C13—C12—C16	120.4 (5)
C3—C2—H2A	121.7	C11—C12—C16A	120.6 (3)
C1—C2—H2A	121.7	C13—C12—C16A	119.1 (3)
C2—C3—C4	121.51 (15)	C14—C13—C12	120.00 (13)
C2—C3—H3A	119.2	C14—C13—H13A	120.0
C4—C3—H3A	119.2	C12—C13—H13A	120.0
C5—C4—C3	121.91 (16)	O2—C14—C13	125.26 (12)
C5—C4—H4A	119.0	O2—C14—C9	115.26 (12)
C3—C4—H4A	119.0	C13—C14—C9	119.48 (12)
C4—C5—C6	117.59 (15)	O2—C15—H15A	109.5
C4—C5—H5A	121.2	O2—C15—H15B	109.5
C6—C5—H5A	121.2	H15A—C15—H15B	109.5
N2—C6—C5	130.48 (13)	O2—C15—H15C	109.5
N2—C6—C1	109.82 (12)	H15A—C15—H15C	109.5
C5—C6—C1	119.70 (13)	H15B—C15—H15C	109.5
N2—C7—N1	113.74 (12)	O3—C16—C12	131.0 (10)
N2—C7—C8	122.82 (11)	O3—C16—H16A	114.5
N1—C7—C8	123.43 (11)	C12—C16—H16A	114.5
O1—C8—C7	108.39 (10)	O3A—C16A—C12	129.3 (5)
O1—C8—H8A	110.0	O3A—C16A—H16B	115.4
C7—C8—H8A	110.0	C12—C16A—H16B	115.4
O1—C8—H8B	110.0		
			
C7—N1—C1—C2	179.33 (14)	O1—C9—C10—C11	179.95 (13)
C7—N1—C1—C6	−0.62 (14)	C14—C9—C10—C11	−0.1 (2)
N1—C1—C2—C3	−179.16 (14)	C9—C10—C11—C12	0.9 (2)
C6—C1—C2—C3	0.8 (2)	C10—C11—C12—C13	−0.9 (2)
C1—C2—C3—C4	−1.0 (2)	C10—C11—C12—C16	177.9 (6)
C2—C3—C4—C5	0.1 (3)	C10—C11—C12—C16A	−179.9 (4)
C3—C4—C5—C6	1.0 (3)	C11—C12—C13—C14	0.0 (2)
C7—N2—C6—C5	−179.88 (14)	C16—C12—C13—C14	−178.8 (6)
C7—N2—C6—C1	−0.13 (14)	C16A—C12—C13—C14	179.0 (4)
C4—C5—C6—N2	178.55 (14)	C15—O2—C14—C13	0.1 (2)
C4—C5—C6—C1	−1.2 (2)	C15—O2—C14—C9	−179.54 (13)
N1—C1—C6—N2	0.47 (14)	C12—C13—C14—O2	−178.79 (12)
C2—C1—C6—N2	−179.48 (12)	C12—C13—C14—C9	0.8 (2)
N1—C1—C6—C5	−179.75 (12)	O1—C9—C14—O2	−1.17 (17)
C2—C1—C6—C5	0.3 (2)	C10—C9—C14—O2	178.85 (12)
C6—N2—C7—N1	−0.28 (15)	O1—C9—C14—C13	179.18 (11)
C6—N2—C7—C8	179.19 (12)	C10—C9—C14—C13	−0.81 (19)
C1—N1—C7—N2	0.59 (15)	C11—C12—C16—O3	176.2 (10)
C1—N1—C7—C8	−178.88 (12)	C13—C12—C16—O3	−4.9 (16)
C9—O1—C8—C7	−177.26 (11)	C16A—C12—C16—O3	50 (19)
N2—C7—C8—O1	174.58 (12)	C11—C12—C16A—O3A	−3.5 (10)
N1—C7—C8—O1	−6.00 (18)	C13—C12—C16A—O3A	177.5 (6)
C8—O1—C9—C10	2.33 (19)	C16—C12—C16A—O3A	51 (20)
C8—O1—C9—C14	−177.65 (11)		

### Hydrogen-bond geometry (Å, °)

**Table:**

*D*—H···*A*	*D*—H	H···*A*	*D*···*A*	*D*—H···*A*
O4—H4WB···O5	0.84 (2)	1.98 (2)	2.809 (2)	170 (2)
O4—H4WA···O5^i^	0.87 (2)	2.04 (2)	2.880 (2)	162 (2)
O5—H5WB···N2	0.85 (1)	1.96 (2)	2.8003 (16)	171 (2)
O5—H5WA···O7^ii^	0.85 (2)	1.94 (2)	2.7882 (19)	171.(2)
O6—H6WA···O4^iii^	0.85 (2)	2.16 (2)	2.996 (2)	171 (3)
O6—H6WB···O2	0.85 (2)	2.40 (2)	3.187 (3)	154 (3)
O7—H7WB···O4^iv^	0.84 (2)	2.01 (2)	2.844 (2)	174 (2)
O7—H7WA···O3A	0.81 (2)	1.98 (2)	2.721 (3)	151 (2)
N1—H1N···O6	0.83 (1)	2.02 (1)	2.8152 (19)	160.(2)

Symmetry codes: (i) −*x*, −*y*+2, −*z*+1; (ii) *x*, *y*+1, *z*+1; (iii) −*x*, −*y*+1, −*z*+1; (iv) −*x*+1, −*y*+1, −*z*.

### Table 2 Cg···Cg π stacking interactions, Cg1, Cg2 and Cg3 are the centroids of rings N1/C1/C6/N2/C7, C1-C6 and C9-C14, respectively; [Symmetry codes: (i) -x,1-y, 1-z; (ii) 1-x, 1-y, 1-z ]

**Table:**

*Cg*X···*Cg*Y	Cg···Cg (Å)	CgX···Perp (Å)	CgY···Perp (Å)
Cg1···Cg3^i^	3.6104 (9)	-3.4276 (5)	-3.4041 (5)
Cg1···Cg3^ii^	3.6288 (9)	3.3426 (5)	3.4101 (5)
Cg2···Cg3^i^	3.9167 (10)	-3.4841 (6)	-3.4068 (5)
